# Pregnancy Cholesterol Metabolism Markers and the Risk of Gestational Diabetes Mellitus: A Nested Case-Control Study

**DOI:** 10.3390/nu15173809

**Published:** 2023-08-31

**Authors:** Yan Li, Yuanjue Wu, Yanyan Ge, Shanshan Huang, Yang Yang, Zhen Zhang, Ningning Cui, Junan Yan, Yonggang Li, Ping Luo, Liping Hao, Guoping Xiong, Xuefeng Yang

**Affiliations:** 1 Department of Nutrition and Food Hygiene, Hubei Key Laboratory of Food Nutrition and Safety, MOE Key Laboratory of Environment and Health, School of Public Health, Tongji Medical College, Huazhong University of Science and Technology, 13 Hangkong Road, Wuhan 430030, China; 2 Shenzhen Center for Chronic Disease Control, Shenzhen 518020, China; 3 School of Public Health, Guangzhou Medical University, Guangzhou 511436, China; 4 Hubei Provincial Key Laboratory for Applied Toxicology, Hubei Provincial Center for Disease Control and Prevention, Wuhan 430079, China; 5 Department of Obstetrics and Gynecology, The Central Hospital of Wuhan, Wuhan 430014, China

**Keywords:** gestational diabetes mellitus, cholesterol metabolism marker, pregnant women, nested case–control study

## Abstract

This study aims to determine the association of pregnancy cholesterol metabolism markers with gestational diabetes mellitus (GDM) risk. We performed a nested case–control study in the Tongji Birth Cohort. GDM was diagnosed according to the 75 g 2 h oral glucose tolerance test (OGTT) at 24–28 gestational weeks. Nine cholesterol metabolism markers were detected using gas chromatography-mass spectrometry. Conditional logistic regression models were conducted. A total of 444 pregnant women were matched in a 1:2 ratio. The cholestanol_TC_ and β-sitosterol_TC_ in cholesterol absorption markers presented negative associations with the risks of GDM (adjusted OR: 0.77, 95% CI: 0.61–0.96; adjusted OR: 0.80, 95% CI: 0.64–1.00). The desmosterol_TC_ in cholesterol synthesis markers were positively associated with the risks of GDM (adjusted OR: 1.25, 95% CI: 1.00–1.56), similar in the ratios of cholesterol synthesis to absorption markers. After adjustment for insulin or HOMA-IR, these effects were reduced. In conclusion, higher cholesterol synthesis and lower cholesterol absorption marker levels in the first pregnancy are associated with a higher risk of GDM, and insulin resistance may play a vital role in this association.

## 1. Introduction

Gestational diabetes mellitus (GDM) is defined as glucose intolerance diagnosed for the first time during pregnancy. It is one of the most common complications that affects approximately 14% of pregnant women worldwide [1]. GDM adversely affects both mothers’ and their offspring’s short-term and long-term health, particularly increasing the incidence of pre-eclampsia, cesarean delivery, macrosomia, and elevating the risk of developing type 2 diabetes mellitus (T2DM). Previous studies have demonstrated that lifestyle interventions such as a rational diet and exercise could prevent GDM [2,3].

Cholesterol plays an essential role in many physiological processes, which is obtained from de novo endogenous cholesterol synthesis and intestinal absorption of dietary and biliary cholesterol [4]. Dietary cholesterol comes from animal foods, including red meat, seafood, poultry, eggs, and dairy products [5]. During pregnancy, due to the regulation of hormones, intestinal fat absorption is enhanced, which easily leads to lipid metabolism disorder. On the other hand, to ensure adequate intake of high-quality protein and micronutrients, animal food intake during pregnancy increases following Chinese traditional customs, resulting in higher cholesterol intake [6]. The previous studies conducted by our research group have demonstrated that the increase in dietary cholesterol intake increased the risk of GDM [6,7]. However, dietary cholesterol is only a part of the source of cholesterol in the body; the relationship between cholesterol synthesis and absorption and GDM risk needs further study. 

It has been proven that serum levels of non-cholesterol sterols could be used as surrogate markers of cholesterol synthesis and absorption [8,9]. These markers can reflect cholesterol metabolism and are often utilized in preventing and treating cardiovascular diseases due to their correlation with blood lipid levels [10,11,12,13]. As for T2DM, one case–control study found that patients with T2DM had metabolic characteristics of low absorption markers and high synthesis of cholesterol markers [10]. In recent years, researchers have found that high levels of cholesterol synthetic markers and low levels of cholesterol absorption markers could be related to new-onset T2DM and may be superior to genetic markers alone [14,15]. In a Finland cohort study, genetic variations in ATP binding cassette subfamily G member 8 (ABCG8) were associated with fasting blood glucose (FBG) levels but did not foresee the risk of T2DM, whereas cholesterol metabolism markers were significantly associated with hyperglycemia and T2DM [15]. However, the studies on the relationship between cholesterol metabolism markers and GDM are limited [16]. Thus, the aim of the present study was to determine the association of pregnancy cholesterol metabolism markers with GDM risk among Chinese pregnant women in a nested case–control study.

## 2. Materials and Methods

### 2.1. Study Participants

We performed a nested case–control study among participants of the Tongji Birth Cohort (TJBC), an ongoing population-based prospective cohort study to identify the effects of maternal diet, lifestyle, and other factors on the health of pregnant women and their offspring. The TJBC was established in March 2018 and healthy pregnant women prior to 16 weeks of gestation were enrolled in this cohort when they went to the hospital for their first antenatal visit in one of three public hospitals in Wuhan. All participants had completed an interview-administrated questionnaire when recruited. This study was carried out in accordance with the Declaration of Helsinki and approved by the Ethics Review Committee of Tongji Medical College, Huazhong University of Science and Technology. Informed written consent was obtained from all participants upon recruitment.

In this study, we included the participants who were drawn blood samples at recruitment and performed the oral glucose tolerance test (OGTT) at 24–28 weeks to diagnose GDM. Women were excluded if they reported multiple pregnancies, a previous history of infectious disease, a previous systemic disease, a previous diagnosis of T2DM or GDM, or assumed drugs interfering with glucose homeostasis and lipid metabolism before pregnancy. Of those participants, 148 cases, in whom GDM developed during this pregnancy, were randomly selected.

Controls were from the participants who did not develop GDM during this pregnancy. Two controls were matched to each case by maternal age, pre-pregnancy BMI, ethnicity, primiparity, and gestational age of blood sampling. Match tolerances that we set are as follows: maternal age is ±5 years old, pregnancy BMI is ±3 kg/m^2^, gestational age of blood sampling is ±4 weeks, and ethnicity and primiparity are the same. Maximizing execution performance was used to match to ensure the comparability between cases and controls.

### 2.2. Laboratory Determinations

Fasting blood samples were drawn from the participants when recruited. Serum samples were separated from the cellular blood components via centrifugation and stored at −80 °C until analysis. Serum levels of total cholesterol (TC), high-density lipoprotein cholesterol (HDL-C), and low-density lipoprotein cholesterol (LDL-C), triglycerides (TG), and FBG were measured by corresponding assay kits (BioSino Bio-Technology & Science Inc., Beijing, China). Insulin concentration was detected using an enzyme-linked immunoassay method by assay kit (Mercodia Inc., Uppsala, Sweden). The insulin resistance index—homeostasis model assessment of insulin resistance (HOMA-IR)—was calculated as follows: HOMA-IR = fasting insulin (mU/L) × fasting glucose (mmol/L)/22.5. Serum cholesterol metabolism markers were assayed by gas chromatography–mass spectrometry (GC–MS) (Agilent, Santa Clara, CA, USA, 7890A-5975C) with 5a-cholestane as the internal standard. Cholesterol was also assayed by GC–MS in the same run as markers with 5β-Cholestan-3α-ol as the internal standard. Absolute cholesterol metabolism markers were expressed in concentrations (mg/L), and relative cholesterol metabolism markers were expressed in ratios to cholesterol (µmol/mmol) or ratios of cholesterol synthesis markers to absorption markers (µmol/µmol).

### 2.3. Outcome Assessment

All the participants routinely completed a 75 g 2 h oral glucose tolerance test (OGTT) during 24–28 weeks. The OGTT was performed in the morning after the patient had completed an overnight fast of at least 8 h. An amount of 0 h fasting blood glucose (0 h FBG), 1 h, and 2 h post-load blood glucose (1 h, 2 h PBG) levels were measured using an automated biochemical analyzer by professional laboratory technicians. GDM was diagnosed when any of the glucose values met or exceeded the criteria as recommended by the International Association of Diabetes and Pregnancy Study Group (IADPSG) [17]. The criteria were as follows: 0 h FBG ≥ 5.1 mmol/L, 1 h PBG ≥ 10.0 mmol/L, or 2 h PBG ≥ 8.5 mmol/L. These results from a 75 g OGTT were collected from medical records.

### 2.4. Other Variables

A structured questionnaire was completed by trained investigators to collect the demographic and socioeconomic characteristics, anthropometric parameters, and lifestyles of the participants at baseline enrollment. The information included maternal age, educational level, average personal income, ethnicity, pre-pregnancy weight, height, family history of the specific disease, parity, physical activity, smoking habit, drinking habit, and so on. Maternal age was categorized as <25, 25–29, 30–34, and ≥35 years old. Ethnicity was categorized as Han Chinese and others. Education level was divided into ≥16 years (bachelor’s degree or above) or <16 years. Per capita monthly income was divided into four categories: ≤2999, 3000–4999, 5000–9999, and ≥10,000 CNY. Pre-pregnancy body mass index (BMI) was divided into four categories according to the BMI classification criteria suitable for Chinese people: <18.5, 18.5–23.9, 24–27.9, and ≥28 kg/m^2^. Smoking (or drinking) habit was defined as smoking (or drinking) no less than three times per week before pregnancy. Physical activity was defined as regular physical activity during pregnancy. Family history of a specific disease was defined as a condition in the father or mother of the participant that was diagnosed by a hospital to meet the diagnostic criteria for the disease, including a family history of obesity, diabetes, dyslipidemia, and hypertension. Smoking habits, drinking habits, physical activity, and other covariates including primiparity, family history of a specific disease, and pregnancy physical activity were treated as dichotomized variables (yes/no).

### 2.5. Statistical Analysis

Descriptive statistics for continuous variables were presented as mean ± SD and for categorical variables were shown as frequencies and percentages. Comparisons of characteristics between GDM cases and controls were made using t-tests for continuous variables and chi-square tests for categorical variables. Log-transformed variables were used during analysis to correct for their skewed distributions for serum cholesterol metabolism markers and biochemical indicators if they have right-skewed distributions.

Pearson product-moment correlation coefficients were used to evaluate the correlation between cholesterol metabolism markers and biochemical indicators. Conditional logistic regression analyses were used to assess the association between the serum cholesterol metabolism markers levels and the risk of GDM, presented as odds ratios (ORs) with 95% confidence intervals (CIs). General linear models were conducted to examine the association of cholesterol metabolism markers with 0 h PBG, 1 h PBG, and 2 h PBG, and the results were presented as coefficients (*β*) with 95% CIs. Covariates that related to cholesterol metabolism markers and GDM reported in the previous literature except matching variables were chosen as potential confounders in multivariable analyses. Multivariate models were as follows: Model 1 was a crude model; Model 2 was adjusted for education, average personal income, family history of diabetes, family history of obesity, smoking before pregnancy, drinking before pregnancy, and leisure-time physical activity; Model 3 was adjusted for Model 2 + insulin level at baseline; Model 4 was adjusted for Model 2 + HOMA-IR at baseline.

All analyses were performed with the SAS version 9.4 (SAS Institute, Cary, NC, USA). A two-sided α of less than 0.05 was considered statistically significant.

## 3. Results

### 3.1. Characteristics of the Participants

Characteristics of the participants are presented in Table 1, including 148 GDM cases and 296 controls. As per the matching criteria, maternal age, pre-pregnancy BMI, ethnicity, primiparity, and gestational age of blood samples were comparable between GDM cases and controls. The average age of the participants was 30.3 years, and the average pre-pregnancy BMI was 21.8 kg/m^2^. GDM cases and controls did not differ in terms of education, average personal income, gravity, family history of diabetes, family history of obesity, smoking and drinking before pregnancy, and leisure-time physical activities.

### 3.2. Serum Biochemical Indicators and Cholesterol Metabolism Markers Concentrations of the Participants

Serum biochemical indicators and cholesterol metabolism markers concentrations of the participants are displayed in Table 2. TG concentration, insulin concentration, and HOMA-IR were significantly higher in GDM cases than in controls and HDL-C concentration was significantly lower in GDM cases than in controls. Other biochemical indicator levels did not significantly differ between the two groups.

Serum concentrations of cholestanol and β-Sitosterol, as well as cholestanol_TC_ and β-Sitosterol_TC_, reflecting cholesterol absorption, were significantly lower in GDM cases than in controls. Additionally, serum desmosterol_TC_ and lathosterol_TC_, reflecting cholesterol synthesis, were significantly higher in GDM cases. The Δ8-Cholestenol_TC_ of the GDM case was almost noticeably higher (*p*-value = 0.07). Most ratios of lathosterol to cholesterol absorption markers, including cholestanol, campesterol, and β-Sitosterol, were significantly higher in GDM cases, with the exception of stigmasterol. Generally, absolute/relative cholesterol synthetic marker levels were higher, and absolute/relative cholesterol absorption marker levels were lower in the GDM cases than in the controls.

### 3.3. Correlations between Cholesterol Metabolism Markers and Biochemical Indicators

**Figure 1:**
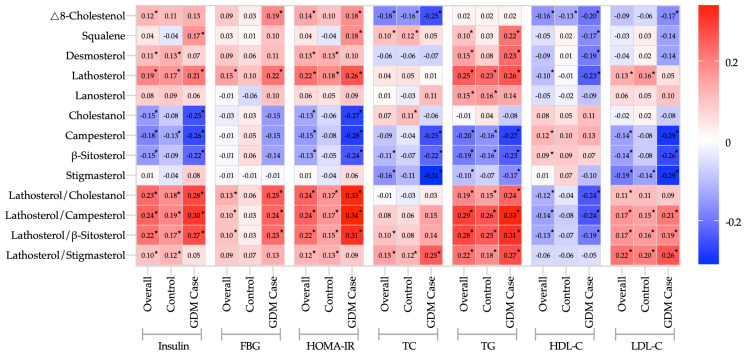
Heatmap of correlation coefficients between serum cholesterol metabolism markers and insulin, FBG, HOMA-IR, and lipid profiles. Pearson product-moment correlation coefficient and relative cholesterol synthesis/absorption markers (μmol/mmol of cholesterol) were log-transformed during analysis to correct for their skewed distributions. * *p*-value < 0.05.

Figure 1 exhibits the heatmap of correlations between cholesterol metabolism markers and biochemical indicators. Serum insulin correlated positively with the relative cholesterol synthesis markers (Δ8-cholestenol_TC_, desmosterol_TC_, lathosterol_TC_, and lanosterol_TC_) (*r* = 0.11–0.19) and the ratios of lathosterol to all absorption markers (*r* = 0.10–0.24) in overall participants, and negatively with the relative cholesterol absorption markers (cholestanol_TC_, campesterol_TC_, and β-sitosterol_TC_) (*r* = −0.18–−0.15). Similar effects occurred in HOMA-IR, FBG, TG, TC, and LDL-C.

Generally, the HDL-C was inversely correlated with cholesterol synthesis markers (Δ8-cholestenol_TC_, desmosterol_TC_, and lathosterol_TC_) and the ratios of lathosterol to absorption markers, and significantly correlated with campesterol_TC_ and β-sitosterol_TC_ in overall participants. 

Comparable results were observed in both the GDM cases and the controls, with a particularly pronounced effect in the GDM cases.

### 3.4. Association between Cholesterol Metabolism Markers and the Risk of GDM

Table 3 shows the associations between maternal cholesterol metabolism markers and the risks of GDM. With respect to cholesterol synthesis markers, they showed positive associations with the risk of GDM. In Model 1, desmosterol_TC_ and lathosterol_TC_ were associated with the risks of GDM. With one SD of log-transformed, the ratios increased, the *OR*s for GDM were 1.24 (95% CI: 1.00–1.54) and 1.26 (95% CI: 1.01–1.59), respectively. After adjustment via Model 2, although the effect of lathosterol_TC_ was slightly reduced, it presented significantly (*p*-value = 0.047), and the effect of desmosterol_TC_ remained. Further adjusted for insulin levels or HOMA-IR, via Model 3 or 4, the clues of those two effects sequentially reduced and ceased to be significant. Despite the large effect size of Δ8-cholestenol, the significant association was still unseen in every model.

In addition, negative associations appeared between cholesterol absorption markers and the risk of GDM. The ratios of cholestanol in relative cholesterol absorption markers stayed inversely associated with the risks of GDM, no matter whether adjusted for other covariates, with the corresponding crude *OR*s and three adjusted *OR*s being 0.76 (95% CI: 0.61–0.96), 0.77 (95% CI: 0.61–0.96), 0.79 (95% CI: 0.63–1.00), and 0.79 (95% CI: 0.63–1.00). Similarly, adverse associations existed between β-sitosterol_TC_ and the risks of GDM in Model 1 (*OR*: 0.80, 95% *CI*: 0.64–1.00) and Model 2 (*OR*: 0.80, 95% *CI*: 0.64–1.00). With further adjustment of insulin or HOMA-IR, the effect weakened to be marginally significant (*p*-value = 0.07).

As for the ratios of cholesterol synthesis to absorption markers, one SD of log-transformed ratios of lathosterol to cholestanol, lathosterol to campesterol, and lathosterol to β-sitosterol increase were associated with the increased GDM risks in the Model 1 and Model 2, with the adjusted ORs presented as 1.36 (95% CI: 1.08–1.72), 1.32 (95% CI: 1.04–1.68), and 1.36 (95% CI: 1.07–1.73), respectively. Further adjusted for insulin levels or HOMA-IR, the associations of the ratios of lathosterol to cholesterol absorption markers of the risks of GDM remained almost significant.

The glucose levels of OGTT also presented comparable findings, displayed in Supplemental Table S1. Especially 0 h PBG, we found it had a significantly positive association with relative cholesterol synthesis markers and the ratios of cholesterol synthesis to absorption markers, such as Δ8-cholestenol_TC_, lathosterol_TC,_ and lathosterol to absorption markers. The negative association between absorption markers and 0 h PBG existed. These positive and negative associations seemed stable whether adjusted for other covariates. With regard to 1 h PBG and 2 h PBG, the overall effects were similar, but the results were hardly significant.

## 4. Discussion

To our knowledge, this is the first nested case–control study that focused on the associations between serum cholesterol metabolism marker levels in the first trimester and the risks of GDM. Our findings demonstrated a strong inverse relationship between elevated serum cholesterol absorption markers and the risk of GDM, while a positive association was observed between higher serum cholesterol synthesis markers and the risk of GDM. An increase in the ratios of cholesterol synthesis to absorption markers was associated with an elevated risk of GDM. Additionally, after adjustment for insulin levels or HOMA-IR, the associations remained significant except that between cholesterol synthesis markers and GDM. Furthermore, the results indicated that higher serum cholesterol synthesis markers and lower serum cholesterol absorption markers could increase FBG.

The present study found that, compared to the control group, the ratios of cholesterol absorption markers (cholestanol and β-sitosterol) to TC were significantly lower and the ratios of cholesterol synthesis markers (lathosterol and desmosterol) to TC were significantly higher in the GDM group. This suggests that pregnant women with GDM have the trait of high cholesterol synthesis efficiency and low cholesterol absorption efficiency in the first trimester, which is basically consistent with the prior study conducted in the obese Finnish population [16]. Miettinen et al. observed that women with GDM had higher levels of Δ8-cholestenol and a higher ratio of lathosterol to β-sitosterol than the control group, indicating that GDM women were characterized by high cholesterol synthesis efficiency. However, this study did not observe any significant differences in cholesterol absorption markers. These might be the causes of these observed differences: First, the participants enrolled in the Finnish study were all obese women with BMI >30 kg/m^2^, and there were significant differences in weight and BMI between GDM and control groups. Previous studies have demonstrated that one of the main factors affecting cholesterol metabolism is obesity [18,19]. In distinct populations with varying glucose metabolism, BMI was positively correlated with the efficiency of cholesterol synthesis. Effective weight loss in patients with T2DM can also improve the absorption efficiency of cholesterol, indicating that obesity may be a confounding factor. BMI was matched in our nested case–control study, which nicely reduced the confounding effect of obesity. Second, the larger sample size of our study is more reliable in reflecting the association between cholesterol metabolism markers and GDM, compared with the small sample size of the Finnish study (22 in the GDM group and 30 in the control group).

The findings of this study were consistent with previous research on the association between cholesterol metabolism markers and T2DM. While patients with type 1 diabetes tend to have high cholesterol absorption and low synthesis, those with T2DM typically have high synthesis and low absorption [18,20,21]. Some researchers have explored the relationship between glucose metabolism and cholesterol metabolism markers via cross-sectional or case–control studies, which have found that cholesterol metabolism varied among individuals with different levels of glucose and glucose tolerance [18,22,23,24]. In cohort or RCT studies, Cederberg and de Mello discovered that cholesterol synthesis markers could be related to the increase in the risk of hyperglycemia and T2DM, while cholesterol absorption markers could be related to the decrease in the risk of hyperglycemia and T2DM [10,15]. However, after adjustment for insulin, the association was not significant, suggesting that insulin resistance may be one of the main mechanisms linking glucose and cholesterol metabolism. It has been demonstrated that insulin resistance has a negative correlation with cholesterol absorption, as well as a positive correlation with cholesterol synthesis [14,25,26,27]. Our study similarly found that insulin levels and HOMA-IR in the first trimester were positively correlated with cholesterol synthesis markers and the ratio of synthesis markers to absorption markers, and negatively correlated with cholesterol absorption markers. In contrast, the relationship between FBG and cholesterol metabolism markers was not significant, indicating that the insulin sensitivity of GDM pregnant women in the first trimester had changed while FBG had not increased significantly. The decrease in insulin sensitivity could partly explain the relationship between cholesterol metabolism and the occurrence of GDM, although the specific mechanism requires further clarification.

In addition to the relationship between cholesterol metabolism and glucose metabolism mentioned above, it was also important to consider the relationship between these cholesterol metabolism markers and blood lipid indicators. Different population studies have found that TC is positively correlated with LDL-C levels and cholesterol absorption efficiency, and negatively correlated with cholesterol synthesis efficiency [28,29,30]. In this study, a cholesterol synthetic marker (Δ8-cholestenol) showed a negative correlation with TC in both groups and a positive association between cholestanol and TC in the control group, consistent with our earlier findings. However, not all studies supported this conclusion [31]. Simonen only observed the association between serum TC and cholesterol absorption markers in non-diabetic patients in the obese population [14]. Additionally, cholestanol, which was the most commonly used index to evaluate cholesterol absorption efficiency, was not found to be associated with serum TC [32]. We also found that HDL-C levels were negatively correlated with synthetic markers and positively correlated with absorption markers, which aligned with the findings of the relationship between HDL-C levels and absorption efficiency in Gylling’s research [32]. Animal experiments have also shown that when cholesterol excretion was inhibited, plasma phytosterol levels were reduced and cholesterol synthesis efficiency was up-regulated, leading to lower cholesterol absorption and lower HDL-C levels [33]. However, it was still unclear which of these variables (cholesterol absorption markers, synthetic markers, lipids, FBG, and insulin levels) was the main regulatory variable, and how they interacted. Rigorous animal experiments and human metabolic tests are necessary to explain their mechanisms.

The main strength of this study was the nested case–control study design, which could allow us to diminish possible confounding bias caused by pre-pregnancy BMI, age, and other variables, and provide strong evidence on the association between cholesterol metabolism markers and GDM. Furthermore, we hypothesized a potential role for insulin in the relationship between cholesterol metabolism and GDM. However, this study still had some limitations. First, we only measured cholesterol metabolism markers in the first trimester of pregnancy and did not monitor their dynamic changes during pregnancy. Second, despite the fact that we took into account a number of GDM risk factors, we were still unable to completely rule out the possibility of residual confounding by other unmeasured factors. Third, more research is required to delve into the molecular processes behind the relationship between cholesterol metabolic indicators and GDM, as the observational features of the current study were unable to illuminate them.

## 5. Conclusions

In this nested case–control study, the results showed that cholesterol absorption marker levels were significantly negatively associated with GDM risk, and synthetic marker levels and their ratios to absorption markers were significantly positively associated with GDM risk. After adjustment for insulin or HOMA-IR, these effects are reduced. High cholesterol synthesis and low cholesterol absorption in first pregnancy may be high-risk factors for GDM, and insulin resistance may play an important role in this association. This study has elucidated the relationship between markers and GDM, thereby contributing novel insights and perspectives to the field of GDM prevention and treatment.

## Figures and Tables

**Table 1:** Characteristics and outcome variables of the participants in a nested case–control study.

	Overall (*n* = 444)	Control (*n* = 296)	GDM Case (*n* = 148)	*p*-Value ^†^
Characteristics				
Gestational age of serum sample (week)	13.9 ± 2.0	13.9 ± 2.1	13.9 ± 2.0	0.69
Gestational age at OGTT (week)	25.3 ± 1.6	25.3 ± 1.3	25.5 ± 2.0	0.07
Age (y)	30.3 ± 3.3	30.1 ± 3.1	30.7 ± 3.7	0.22
<25	10 (2.3)	7 (2.4)	3 (2.0)	0.49
25~	222 (50.0)	152 (51.4)	70 (47.3)	
30~	171 (38.5)	114 (38.5)	57 (38.5)	
≥35	41 (9.2)	23 (7.8)	18 (12.2)	
Height (cm)	161.4 ± 4.9	161.2 ± 4.9	161.9 ± 4.8	0.11
Pre-pregnancy weight (kg)	56.9 ± 8.1	56.4 ± 8.1	57.8 ± 8.1	0.08
Pre-pregnancy BMI (kg/m^2^)	21.8 ± 2.8	21.7 ± 2.8	22.0 ± 2.8	0.14
<18.5	45 (10.1)	30 (10.1)	15 (10.1)	0.77
18.5~	311 (70.0)	210 (70.9)	101 (68.2)	
24.0~	70 (15.8)	46 (15.5)	24 (16.2)	
≥28	18 (4.1)	10 (3.4)	8 (5.4)	
Ethnicity (Han Chinese)	444 (100.0)	296 (100.0)	148 (100.0)	1.00
Education (schooling years≥ 16y)	225 (50.7)	150 (50.7)	75 (50.7)	1.00
Average personal income (CNY/month)				
≤2999	8 (1.8)	6 (2.0)	2 (1.4)	0.13
~4999	76 (17.1)	43 (14.6)	33 (22.2)	
~9999	236 (53.2)	157 (53.0)	79 (53.4)	
≥10,000	124 (27.9)	90 (30.4)	34 (23.0)	
Gravity (times)				
1	247 (55.6)	160 (54.0)	87 (58.8)	0.60
2	120 (27.0)	84 (28.4)	36 (24.3)	
≥3	77 (17.4)	52 (17.6)	25 (16.9)	
Primiparity (yes)	333 (75.0)	222 (75.0)	111 (75.0)	1.00
Family history of diabetes (yes)	49 (11.0)	31 (10.5)	18 (12.2)	0.59
Family history of obesity (yes)	2 (0.5)	2 (0.7)	0 (0.0)	0.56
Family history of hypertension (yes)	142 (32.0)	90 (30.4)	52 (35.1)	0.31
Family history of hyperlipidemia (yes)	16 (3.6)	9 (3.0)	7 (4.7)	0.37
Smoking before pregnancy (yes)	10 (2.3)	4 (1.4)	6 (4.1)	0.07
Drinking before pregnancy (yes)	18 (4.1)	12 (4.1)	6 (4.1)	1.00
Leisure-time physical activity (yes)	197 (44.6)	123 (41.9)	74 (50.0)	0.09
Outcome variables				
0 h FBG (mmol/L)	4.7 ± 0.4	4.5 ± 0.3	4.9 ± 0.4	<0.001
1 h PBG (mmol/L)	8.2 ± 1.7	7.6 ± 1.3	9.5 ± 1.6	<0.001
2 h PBG (mmol/L)	7.1 ± 1.4	6.6 ± 1.1	8.2 ± 1.4	<0.001

Continuous variables were presented as mean ± SD, and categorical variables were shown as *n* (%). ^†.^*p*-values were from *t*-tests for continuous data and from chi-square tests for categorical data.

**Table 2:** Serum and lipoprotein cholesterol, serum triglyceride, insulin, glucose, HOMA-IR, and markers of cholesterol metabolism concentration of the participants.

	Overall (*n* = 444)	Control (*n* = 296)	GDM Case (*n* = 148)	*p*-Value ^†^
TC ^a^ (mmol/L)	4.87 ± 1.14	4.87 ± 1.16	4.89 ± 1.08	0.60
HDL-C (mmol/L)	2.10 ± 0.48	2.13 ± 0.47	2.03 ± 0.48	0.02
LDL-C (mmol/L)	2.47 ± 0.73	2.45 ± 0.75	2.53 ± 0.67	0.17
TG (mmol/L)	1.68 ± 0.74	1.63 ± 0.70	1.78 ± 0.81	0.04
FBG (mmol/L)	4.25 ± 1.02	4.17 ± 0.99	4.42 ± 1.06	0.052
Insulin (mU/L)	7.52 ± 8.10	6.82 ± 6.44	8.90 ± 10.56	<0.01
HOMA-IR	1.54 ± 2.29	1.36 ± 1.83	1.89 ± 2.99	<0.01
Absolute cholesterol metabolism markers				
Cholesterol synthesis markers (mg/L)				
Δ8-Cholestenol	0.53 ± 0.16	0.53 ± 0.16	0.55 ± 0.18	0.20
Squalene	0.24 ± 0.40	0.23 ± 0.22	0.26 ± 0.61	0.42
Desmosterol	0.43 ± 0.27	0.41 ± 0.11	0.46 ± 0.44	0.18
Lathosterol	3.30 ± 1.56	3.24 ± 1.60	3.41 ± 1.49	0.17
Lanosterol	0.06 ± 0.03	0.06 ± 0.03	0.06 ± 0.03	0.66
Cholesterol absorption markers (mg/L)				
Cholestanol	2.14 ± 0.90	2.20 ± 0.93	2.02 ± 0.82	0.02
Campesterol	2.17 ± 1.12	2.23 ± 1.17	2.05 ± 1.01	0.10
β-Sitosterol	2.41 ± 1.30	2.49 ± 1.32	2.27 ± 1.24	0.02
Stigmasterol	0.10 ± 0.08	0.10 ± 0.06	0.10 ± 0.10	0.88
Relative cholesterol metabolism markers				
Cholesterol synthesis markers (µmol/mmol of TC ^b^)				
Δ8-Cholestenol_TC_^c^	0.29 ± 0.09	0.28 ± 0.08	0.30 ± 0.10	0.07
Squalene_TC_^c^	0.14 ± 0.21	0.13 ± 0.10	0.15 ± 0.33	0.63
Desmosterol_TC_^c^	0.23 ± 0.12	0.22 ± 0.05	0.25 ± 0.19	0.047
Lathosterol_TC_^c^	1.75 ± 0.76	1.71 ± 0.75	1.83 ± 0.77	0.04
Lanosterol_TC_^c^	0.03 ± 0.01	0.03 ± 0.01	0.03 ± 0.01	0.88
Cholesterol absorption markers (µmol/mmol of TC ^b^)				
Cholestanol_TC_^c^	1.13 ± 0.42	1.16 ± 0.46	1.06 ± 0.32	0.02
Campesterol_TC_^c^	1.13 ± 0.65	1.16 ± 0.68	1.07 ± 0.59	0.17
β-Sitosterol_TC_^c^	1.22 ± 0.74	1.25 ± 0.77	1.15 ± 0.67	0.046
Stigmasterol_TC_^c^	0.05 ± 0.04	0.05 ± 0.03	0.05 ± 0.05	0.72
Cholesterol synthesis/absorption ratios (µmol/µmol)				
Lathosterol/Cholestanol	1.72 ± 0.92	1.64 ± 0.89	1.87 ± 0.97	<0.01
Lathosterol/Campesterol	1.88 ± 1.20	1.82 ± 1.22	2.00 ± 1.17	0.03
Lathosterol/β-Sitosterol	1.74 ± 1.07	1.65 ± 1.02	1.91 ± 1.14	0.01
Lathosterol/Stigmasterol	48.60 ± 49.35	48.57 ± 53.56	48.67 ± 39.81	0.48

Continuous variables were presented as mean ± SD. ^†^ *p*-values were calculated by using conditional logistic regression, which accounts for the matched nature of the sample. Characteristics were log-transformed during analysis to correct for their skewed distributions. ^a^ TC was analyzed using enzymatic-spectrophotometric methods; ^b^ TC was analyzed using GC–MS methods; ^c^ The markers were relative cholesterol metabolism markers, which were the ratios of cholesterol synthesis/absorption to cholesterol.

**Table 3:** Associations between maternal cholesterol synthesis and absorption markers and GDM risk.

Per 1-SD of Log-Transformed Increase	Model 1		Model 2		Model 3		Model 4	
	OR (95% CI)	*p*-Value	OR (95% CI)	*p*-Value	OR (95% CI)	*p*-Value	OR (95% CI)	*p*-Value
Δ8-Cholestenol_TC_	1.22 (0.98, 1.51)	0.07	1.23 (0.99, 1.54)	0.07	1.18 (0.95, 1.48)	0.14	1.18 (0.94, 1.48)	0.15
Squalene_TC_	0.94 (0.73, 1.21)	0.63	0.95 (0.74, 1.23)	0.71	0.97 (0.75, 1.26)	0.82	0.96 (0.74, 1.25)	0.78
Desmosterol_TC_	1.24 (1.00, 1.54)	0.047	1.25 (1.00, 1.56)	0.047	1.22 (0.98, 1.53)	0.08	1.23 (0.98, 1.54)	0.07
Lathosterol_TC_	1.26 (1.01, 1.59)	0.04	1.27 (1.00, 1.60)	0.047	1.22 (0.96, 1.54)	0.10	1.21 (0.96, 1.53)	0.11
Lanosterol_TC_	0.98 (0.77, 1.24)	0.88	1.01 (0.80, 1.29)	0.91	1.00 (0.78, 1.27)	0.97	1.01 (0.79, 1.29)	0.96
Cholestanol_TC_	0.76 (0.61, 0.96)	0.02	0.77 (0.61, 0.96)	0.02	0.79 (0.63, 1.00)	0.045	0.79 (0.63, 1.00)	0.047
Campesterol_TC_	0.86 (0.69, 1.07)	0.17	0.84 (0.67, 1.04)	0.12	0.85 (0.68, 1.06)	0.16	0.85 (0.68, 1.06)	0.16
β-Sitosterol_TC_	0.80 (0.64, 1.00)	0.046	0.80 (0.64, 1.00)	0.048	0.81 (0.65, 1.01)	0.07	0.81 (0.65, 1.01)	0.07
Stigmasterol_TC_	1.04 (0.84, 1.29)	0.72	1.03 (0.82, 1.29)	0.83	1.06 (0.84, 1.34)	0.64	1.04 (0.83, 1.32)	0.71
Lathosterol/Cholestanol	1.36 (1.09, 1.71)	<0.01	1.36 (1.08, 1.72)	<0.01	1.30 (1.03, 1.64)	0.03	1.29 (1.02, 1.63)	0.03
Lathosterol/Campesterol	1.29 (1.02, 1.62)	0.03	1.32 (1.04, 1.68)	0.02	1.27 (0.99, 1.62)	0.06	1.26 (0.99, 1.61)	0.06
Lathosterol/β-Sitosterol	1.35 (1.07, 1.71)	0.01	1.36 (1.07, 1.73)	0.01	1.31 (1.03, 1.66)	0.03	1.30 (1.02, 1.66)	0.03
Lathosterol/Stigmasterol	1.08 (0.87, 1.35)	0.48	1.10 (0.88, 1.38)	0.41	1.05 (0.83, 1.33)	0.67	1.06 (0.84, 1.33)	0.64

Relative cholesterol synthesis and absorption markers (μmol/mmol of cholesterol) and the ratio of cholesterol synthesis markers to absorption markers (μmol/μmol) were log-transformed during analysis to correct for their skewed distributions. Conditional logistic regression models were used. Matching variables: maternal age, pre-pregnancy BMI, ethnicity, primiparity, and gestational age of the blood sample. The models were as follows: Model 1, crude model; Model 2, adjusted for maternal education, average personal income, family history of diabetes, family history of obesity, smoking before pregnancy, drinking before pregnancy, and leisure-time physical activity; Model 3, adjusted for Model 2 + insulin level at baseline; Model 4, adjusted for Model 2 + HOMA-IR at baseline.

## Data Availability

The data presented in this study are available upon request from the corresponding author. The data are not publicly available due to privacy.

## Supplementary Materials

The following supporting information can be downloaded at https://www.mdpi.com/article/10.3390/nu15173809/s1, Table S1: Associations between maternal cholesterol synthesis and absorption markers and OGTT.
