# Supplementary material for: Pregnancy Cholesterol Metabolism Markers and the Risk of Gestational Diabetes Mellitus: A Nested Case-Control Study
Source: Nutrients. 2023 Aug 31;15(17):3809. doi: 10.3390/nu15173809 (PMC10490088; doi:10.3390/nu15173809)
Supplement: Supplementary file 1 [file nutrients-15-03809-s001.zip › nutrients-2527234-supplementary.pdf]

Table S1 Associations between maternal cholesterol synthesis and absorption markers and OGTT

Per 1-SD of log-transformed increase	0 h FBG (mmol/L)				1 h PBG (mmol/L)				2 h PBG (mmol/L)			
	Model 1		Model 2		Model 1		Model 2		Model 1		Model 2	
	β (95% *CI*)	*P* value	β (95% *CI*)	*P* value	β (95% *CI*)	*P* value	β (95% *CI*)	*P* value	β (95% *CI*)	*P* value	β (95% *CI*)	*P* value
△8-Cholestenol$_{_TC}$	0.06 (0.02, 0.11)	<0.01	0.06 (0.02, 0.11)	<0.01	0.10 (-0.08, 0.29)	0.27	0.09 (-0.09, 0.28)	0.33	0.10 (-0.05, 0.25)	0.20	0.09 (-0.06, 0.25)	0.24
Squalene$_{_TC}$	0.02 (-0.03, 0.07)	0.40	0.02 (-0.03, 0.07)	0.42	0.00 (-0.20, 0.19)	0.96	-0.02 (-0.22, 0.18)	0.82	-0.06 (-0.23, 0.11)	0.49	-0.06 (-0.23, 0.11)	0.46
Desmosterol$_{_TC}$	0.04 (0.00, 0.08)	0.08	0.03 (-0.01, 0.08)	0.12	0.18 (0.00, 0.35)	0.052	0.17 (-0.01, 0.34)	0.07	0.21 (0.06, 0.36)	<0.01	0.21 (0.06, 0.36)	<0.01
Lathosterol$_{_TC}$	0.06 (0.02, 0.11)	<0.01	0.06 (0.01, 0.10)	<0.01	0.12 (-0.06, 0.30)	0.19	0.09 (-0.09, 0.28)	0.32	0.12 (-0.03, 0.28)	0.12	0.10 (-0.05, 0.26)	0.19
Lanosterol$_{_TC}$	0.03 (-0.02, 0.07)	0.26	0.03 (-0.02, 0.08)	0.21	0.02 (-0.18, 0.21)	0.87	-0.01 (-0.20, 0.19)	0.94	0.05 (-0.12, 0.21)	0.56	0.04 (-0.13, 0.21)	0.63
Cholestanol$_{_TC}$	-0.06 (-0.11, -0.02)	0.01	-0.06 (-0.10, -0.01)	0.02	-0.18 (-0.38, 0.01)	0.07	-0.18 (-0.38, 0.02)	0.07	-0.13 (-0.30, 0.03)	0.12	-0.12 (-0.29, 0.05)	0.17
Campesterol$_{_TC}$	-0.08 (-0.12, -0.03)	<0.01	-0.07 (-0.12, -0.03)	<0.01	-0.02 (-0.21, 0.17)	0.81	-0.01 (-0.20, 0.18)	0.89	-0.02 (-0.18, 0.14)	0.79	-0.01 (-0.18, 0.15)	0.86
β-Sitosterol$_{_TC}$	-0.07 (-0.12, -0.03)	<0.01	-0.07 (-0.11, -0.02)	<0.01	-0.05 (-0.24, 0.14)	0.62	-0.04 (-0.23, 0.14)	0.65	-0.03 (-0.19, 0.13)	0.70	-0.02 (-0.18, 0.14)	0.78
Stigmasterol$_{_TC}$	0.00 (-0.04, 0.05)	0.96	-0.01 (-0.05, 0.04)	0.82	0.08 (-0.10, 0.26)	0.40	0.06 (-0.13, 0.24)	0.55	0.09 (-0.06, 0.25)	0.23	0.08 (-0.07, 0.24)	0.29
Lathosterol/Cholestanol	0.08 (0.04, 0.13)	<0.01	0.08 (0.03, 0.12)	<0.01	0.19 (0.00, 0.37)	0.048	0.17 (-0.02, 0.36)	0.08	0.16 (0.01, 0.32)	0.04	0.15 (-0.02, 0.31)	0.08
Lathosterol/Campesterol	0.09 (0.05, 0.13)	<0.01	0.08 (0.04, 0.13)	<0.01	0.09 (-0.09, 0.28)	0.32	0.07 (-0.11, 0.25)	0.46	0.09 (-0.06, 0.25)	0.23	0.08 (-0.08, 0.23)	0.34
Lathosterol/β-Sitosterol	0.08 (0.04, 0.13)	<0.01	0.08 (0.04, 0.12)	<0.01	0.11 (-0.07, 0.29)	0.25	0.09 (-0.10, 0.27)	0.35	0.10 (-0.05, 0.25)	0.21	0.08 (-0.08, 0.24)	0.31
Lathosterol/Stigmasterol	0.04 (-0.01, 0.08)	0.12	0.04 (-0.01, 0.08)	0.10	0.00 (-0.18, 0.19)	0.96	0.01 (-0.18, 0.19)	0.95	-0.01 (-0.16, 0.15)	0.92	-0.01 (-0.17, 0.15)	0.89

Relative cholesterol synthesis and absorption markers (μmol/mmol of cholesterol) and the ratio of cholesterol synthesis markers to absorption markers (μmol/μmol) were log-transformed during analysis to correct for their skewed distributions. General linear models were used. The models were as follows: Model 1, crude model; Model 2, adjusted for maternal education, average personal income, family history of diabetes, family history of obesity, smoking before pregnancy, drinking before pregnancy and leisure-time physical activity.
